# Perceiving the Direction of Articulatory Motion in Point-Light Actions

**DOI:** 10.1371/journal.pone.0115117

**Published:** 2014-12-19

**Authors:** Alex Davila, Ben Schouten, Karl Verfaillie

**Affiliations:** Laboratory of Experimental Psychology, University of Leuven, Leuven, Belgium; Birkbeck, University of London, United Kingdom

## Abstract

Human observers are able to perceive the motion direction of actions (either forward or backward) on the basis of the articulatory, relative motion of the limbs, even when the actions are shown under point-light conditions. However, most studies have focused on the action of walking. The primary purpose of the present study is to further investigate the perception of articulatory motion in different point-light actions (walking, crawling, hand walking, and rowing). On each trial, participants were presented with a forward or backward moving person and they had to decide on the direction of articulatory motion of the person. We analyzed sensitivity (d') as well as response bias (c). In addition to the type of action, the diagnosticity of the available information was manipulated by varying the visibility of the body parts (full body, only upper limbs, or only lower limbs) and the viewpoint from which the action was seen (from frontal view to sagittal view). We observe that, depending on the specific action, perception of direction of motion is driven by different body parts. Implications for the possible existence of a life detector, i.e., an evolutionarily old and innate visual filter that is tuned to quickly and automatically detect the presence of a moving living organism and direct attention to it, are discussed.

## Introduction

The Leuven psychologist Albert Michotte, a pioneer in research on the perception of animate motion, pointed out already several decades ago that “a thorough study of behaviour must take into account the way in which people and animals ‘understand’ the actions of other people and animals, as well as those they perform themselves” [1], p. 34. Indeed, accurate perception and understanding of the actions and intentions of conspecifics is a crucial prerequisite for adequate social interaction [2], [3]. A remarkable demonstration of this ability is the perception of point-light actions [4]: A handful of point lights attached to strategic positions on the body of a moving human body is sufficient to allow the observer to pick up several behaviorally relevant properties both of the moving person and of the action performed (see [5]–[8] for reviews and some historical background; note that, in his seminal paper on biological motion perception, Johansson refers to Michotte's work on the perception of animate motion: “Stimulus patterns representing animals in motion have been rarely studied. Michotte's [1963; originally published in 1946] study of perception of larva motion may be pointed to as an important exception” [4], p. 201.)

Although it has been documented repeatedly that human observers easily identify different types of human (inter)actions under point-light conditions (e.g., [9]–[12]), most studies of biological motion perception focused on the perception of human walking. One aspect that has received attention in the literature is the perception of the direction of walking (forward vs. backward) of a walker moving on a treadmill, in human observers (e.g., [8], [13]–[16]), as well as in nonhuman primates [17]. With this stimulus configuration the global translatory component of motion (common motion) is set to zero and discrimination of the direction of motion can only be based on the articulatory movements of the body parts in relation to each other (relative motion; e.g., [18], [19]).

For instance, in a study of Verfaillie [20], participants had to discriminate between a biological motion walker and a similar distractor. The point-light walker was facing either to the right or to the left and walking either forward or backward. Forward walking figures were identified faster than backward walking figures. (Note that the walking direction was task irrelevant in this study.) In a follow-up study [8] participants had to discriminate between forward and backward walking figures (making perception of walking direction task relevant). Latencies to forward walking displays were again shorter than to backward walking displays (but the difference was not statistically significant).

There are several possible, not necessarily mutually exclusive, reasons to speculate why backward walking might be more difficult to perceive and interpret than forward walking. First, human observers are relatively rarely confronted with backward moving walkers, so familiarity might play a role. It has indeed been suggested repeatedly that stored knowledge of actions (or the identity of the actor) play a pivotal role in action and posture perception (e.g., [21]–[24]). Second, the anatomy of the human body probably is not optimally suited for backward walking. Under the assumption that motor knowledge influences visual perception of human movements (e.g., [25]–[28]), perception of backward movement might therefore be compromised.

The primary aim of the present study is to further investigate the perception of the direction of motion (either forwards or backwards) on the basis of the articulatory, relative motion of the limbs, not only when observers view a walking action but also when perceiving other actions. More specifically, perception of the direction of articulatory motion is studied in walking, crawling, hand walking, and rowing. We use a signal detection paradigm. On each trial, participants are presented with a forward (signal) or backward (noise) moving person and they have to decide on the direction of articulatory motion of the person. We analyze sensitivity (d') as well as response bias (c).

One obvious (and the most important for the present examination) reason for studying direction of motion perception not only when observers view a walking action but also when perceiving other actions has to do with generalizability from findings on walking to other actions. However, in addition to that, there are other, more theoretical, reasons. The perception of the direction of *walking* probably is mainly driven by the movement of the extremities of the moving human body, especially the movement of the feet. Troje and colleagues even have suggested [29]–[34] that during the perception of human locomotion a specialized life detector mechanism is activated, an evolutionarily old and innate visual filter that is tuned to quickly and automatically detect the presence of a moving living organism and direct attention to it. In fact, Michotte [35] already alluded to this possibility: “les mouvements exécutés par l'homme ou l'animal possèdent un caractère special qui les différencie nettement, d'ordinaire, des mouvements des objects inanimés, et qui permet de reconnâitre aisément la présence d'une vie animale, fait capital au point de vue biologique (the movements performed by men or animals have a special nature that differentiates them clearly from the movements of inanimate objects and that allow effortless recognition of the presence of animate life, an important fact from a biological point of view)”.

Because of the presence of gravitational forces, perceived acceleration patterns in the movements of the feet during walking play a prominent role in this process. Research on the perception of the articulatory direction of motion, also in other actions than walking, might lead to a better understanding of this life detector.

Given the evolution of the human species towards upright, bipedal, walking, it is not surprising that perception of motion direction indeed probably is mainly driven by the movements of the lower limbs during the perception of most common forms of human locomotion. However, for other, less common, locomotion styles, it can be expected that the upper limbs also carry important information. In Experiments 1 and 2, participants viewed the traditionally studied action of walking, in which motion of the limbs probably is most diagnostic for direction discrimination. In Experiment 3, observers were presented with a crawling action [36]–[38]. It can be expected that, even though the action is uncommon, for this quadruped mode of locomotion both lower and upper limbs carry information of the direction of articulatory motion. In Experiment 4, we showed subjects an even less familiar action, namely hand walking [39], in which the movements of the arms probably are most diagnostic (and the legs carry no useful information). The action stimulus in Experiment 5 consisted of a rowing action. In contrast to the intransitive (i.e., no accessory devices are necessary for locomotion) actions of walking, crawling, and hand walking, rowing is a transitive action (i.e., motion is realized not by direct contact of the limbs with the ground surface, but indirectly by making use of locomotory tools like a boat). In the case of rowing, direction of articulatory motion probably mainly is signaled by the movements of the upper limbs.

Apart from varying the type of action, a second way in which we manipulated the diagnosticity of different body parts simply consisted of restricting the available stimulus information either to the upper limbs or to the lower limbs (in comparison to a control condition in which the full body was presented) for the different types of actions (see[40] for related research on the role of different body parts in direction discrimination of point-light actions).

Thirdly, perception of movement direction probably varies with the viewpoint from which the point-light stimulus is seen. For instance, the profile orientation of a walker is likely to carry more information than the frontal orientation of a walker. Kuhlmann, Lussanet, and Lappe [13] reported research on the perception of point-light limited-lifetime full body human walkers going either backwards or forwards and shown in different orientations and observed that walking direction could be readily seen in profile and half-profile views, but direction discrimination became very difficult in frontal views.

In sum, we predict that sensitivity to perceive the direction of motion depends on the perceived action, the perceivable body part, and the viewpoint from which the action is seen. We also analyzed response bias, but predictions are less straightforward here. A bias for forward motion when the uncertainty about the direction of motion is increased could be predicted. Indeed, under the latter circumstances observers might be prompted to perceive forward motion (if familiarity and/or motor knowledge influence perception of direction of articulation). In Bayesian terms, the perceptual system integrates available sensory evidence with expectations about the state of the external environment priors [41]–[44]. Prior expectations (e.g., on the basis of familiarity) are expected to bias observer's performance especially when stimulus-driven processing is made more difficult (i.e., by manipulating the figure's in-depth orientation and visible body part).

## Experiment 1

In Experiment 1 we focused on the perception of walking. On each trial, participants were presented with a sagittal 0° view of a point-light walker, facing to the left. (Viewpoint was not yet manipulated in Experiment 1.) They pressed one button when they perceived the figure as walking forward and another button when they perceived the walker as walking backward. Under the assumption that walking direction is carried primarily by information in the lower part of the body, we predicted that performance would be worst when only the upper part of the body was visible and that performance in the condition in which only the diagnostic lower part was visible wouldn't differ much from the full-body condition.

### Method

#### Participants

14 students of psychology at the University of Leuven (KULeuven) (13 women, 1 man, M_age_ = 18.78 years, SD_age_ = 1.03 years) participated in this experiment. All observers had normal or corrected to normal vision and were naive to the purpose of the experiment. The study (and all other experiments reported in the present article) was approved by the Ethical Committee of the Faculty of Psychology and Educational Sciences of the University of Leuven and in accordance with the ethical standards laid down in the 1964 Declaration of Helsinki. Participants provided written informed consent (following the consent procedure approved by the Ethical Committee). A few participants were under 18: 1 participant in Experiment 1 (age 17.78), 1 participant in Experiment 3 (age 17.83), and 1 participant in Experiment 5 (age 17.90). For these participants we did not obtain consent from their parents or legal guardians, because the Ethical Committee of our faculty urges this only for participants under the age of 16. Just before the beginning of the experiment, written instructions were given and practice took place to make sure that participants understood the task and were well prepared for the main task.

#### Stimulus

The stimulus consisted of a point-light human walker in profile orientation going either backwards or forwards. The point-light walker was designed using motion capture data from a real walker and a 3D animation technique [12], [45]. The animation was created with MATLAB for Windows XP to play 60 still images (for one step cycle, consisting of two steps) with a frame refreshing rate of 60 Hz on the screen of a Dell monitor. Each image in the full body version consisted of 13 white dots positioned on the major joints of the walker (the head, two shoulders, two elbows, two wrists, two hips, two knees, and two ankles; radius  = 3 pixels) on a gray background. The walker subtended 4 cm at a viewing distance of 45 cm. Upper body and lower body walkers were generated drawing only the dots corresponding to the upper (head, two shoulders, two elbows, and two wrists) and lower (two hips, two knees, and two ankles) joints of the body, respectively. For the upper and lower body only stimuli, the dots corresponding to the lower and upper body, respectively, were invisible. To generate the animation of backward motion, the sequence of frames was reversed.

#### Procedure and design

The experiment was run in a dimly lit and sound-attenuated lab room. On each trial, participants were presented with a sagittal 0° view of the upper, lower, or full body of a point-light walker, facing to the left. They were instructed to press one button when they perceived the figure as walking forward and another button when they perceived the walker as walking backward. Stimuli were presented for 4.5 sec. After stimulus presentation, a response screen appeared asking whether the stimulus figure was moving forward or backward. Two blocks of trials were administered to each participant. Each block contained 20 trials in each of the 6 conditions (2 directions of motion ×3 types of walkers). Stimuli were presented in a random order. Before the beginning of experiment, the set of all stimuli was shown (named as forward or backward walking) and written instructions were given. Feedback was provided after each trial and after each block of trials (by giving the percentage of correct responses).

#### Data availability

The full data set is available by email request to Alex Davila, who now is at the Pontifical Catholic University of Peru (adavila@pucp.edu.pe).

### Results and Discussion

**Figure 1:**
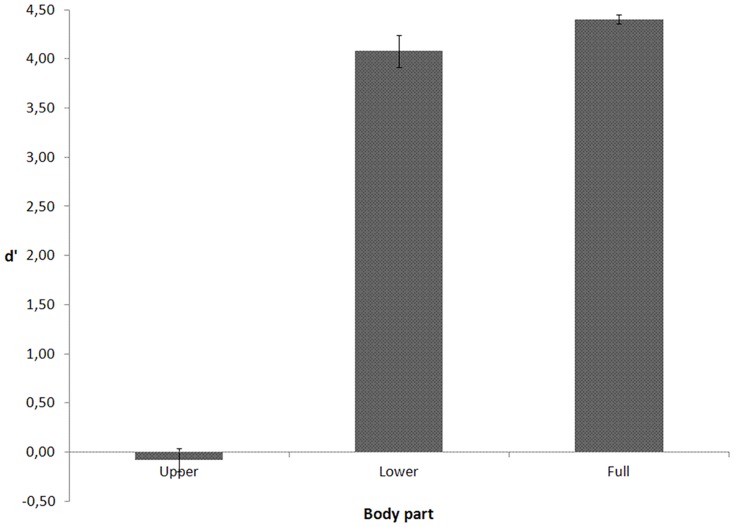
d' (including standard error bars) as a function of visible body part for the walking action in Experiment 1.

A signal detection analysis was performed (classifying forward motion as the “signal” and backward motion as the “noise”). Sensitivity (d') values are depicted in Fig. 1. In a repeated measures one-way analysis of variance (ANOVA) with visible body part (upper, lower, full) as within-subject variable, Mauchly's tests indicated that the assumption of sphericity for that variable had been violated (χ2(2)  = 10.912, p = .004). Therefore, degrees of freedom were corrected using Greenhouse-Geisser estimates of sphericity (ε = .606). The ANOVA yielded a significant main effect of body part, F(1.212, 15.758)  = 470.541, p<.001. Planned comparisons showed that d' for the upper part condition (M = −.079, SE = .114) was lower than d' for the lower part (M = 4.078, SE = .160), p<.001, and the full body conditions (M = 4.402, SE = .046), p<.001. As expected, when only the (less informative) upper body part was visible, discrimination between forward and backward motion was impossible. When the (more informative) lower part was visible, discrimination was equivalent to performance with the full body. It is obvious that, for the walking action, direction discrimination based on the analysis of relative motion is primarily driven by the motion of the lower limbs.

**Figure 2:**
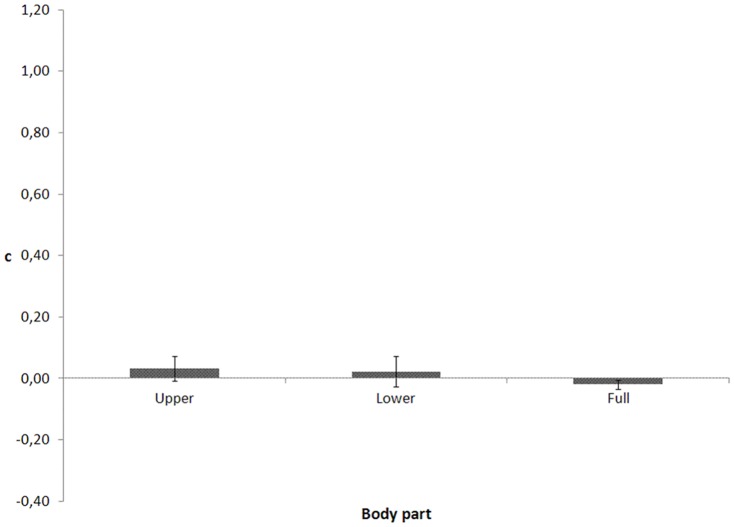
c as a function of visible body part for the walking action in Experiment 1.

Fig. 2 depicts the c values. Mauchly's test indicated that the assumption of sphericity for the body part had not been violated (χ2(2)  = 1.776, p = .411). The ANOVA did not yield a significant main effect of body part, F(2, 26)  = .641, p = .535.

## Experiment 2

In Experiment 2, we again manipulated the available body part information (full, upper, or lower) of a point-light walker. In addition, under the assumption that the diagnosticity of the information signalling walking direction varies with the viewpoint from which the walker is observed, the in-depth orientation of the point-light walker was varied.

We predicted, as in Experiment 1, that performance would be worst when only the upper part of the body was visible and that performance in the condition in which only the lower part was visible would not differ much from the full-body condition. The new prediction in Experiment 2 was that performance would deteriorate in frontal orientation conditions in comparison to the other orientation conditions.

### Methods

#### Participants

14 students of psychology at the KU Leuven with normal vision or corrected to normal vision (12 women, 2 men, M_age_ = 18.57, SD_age_ = .542 years) participated in this experiment. None of them had taken part in the previous experiment.

#### Stimuli

The point-light human walker as used in Experiment 1 was now shown in one of four possible orientations: the 0° profile orientation, the 90° frontal orientation or the 30° or 60° orientation in between. Again, the walker was going either forwards or backwards as moving on a treadmill and the full body, only the upper body, or only the lower body was shown.

#### Procedure and design

As in Experiment 1, participants on each trial had to indicate whether the walker was moving forward or backward. Two blocks of trials were administered to each participant. Each block contained 120 trials consisting of 5 trials for each of the 24 conditions (2 directions of motion ×3 types of walkers ×4 orientations). The sequence of trials within each block was again randomized.

### Results and discussion

**Figure 3:**
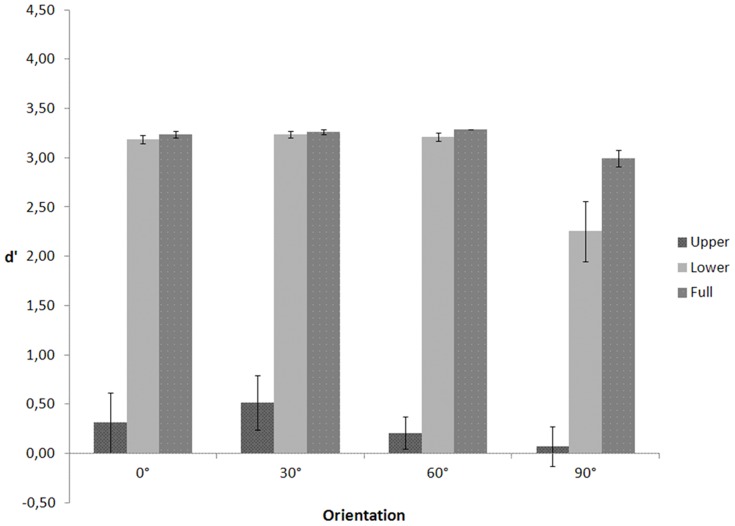
d' as a function of visible body part and figure orientation for the walking action in Experiment 2.

In a repeated measures ANOVA on d' (see Fig. 3) with visible body part (upper, lower, full) and depth orientation (0°, 30°, 60°, and 90°) as within-subject variables, Mauchly's tests indicated that the assumptions of sphericity for the effect of body part (χ2(2)  = 11.261, p = .004), orientation (χ2(5)  = 17.357, p = .004), and the interaction between body part and orientation (χ2(20)  = 84.215, p<.001) were violated. Therefore, degrees of freedom were corrected using Greenhouse-Geisser estimates of sphericity for the body part (ε = .622), orientation (ε = .555), and the interaction (ε = .439). The ANOVA yielded a significant main effect of body part, F(1.243, 16.162)  = 237.005, p<.001, and orientation, F(1.665, 21.650)  = 6.472, p = .009. The interaction between body part and orientation, F(2.635, 34.253)  = 2.156, p = .118 was not significant. Planned comparisons showed that d' for the upper part conditions (M = .277, SE = .159) was lower than d' for the lower part (M = 2.972, SE = .085, p<.001) and full body conditions (M = 3.196, SE = .022, p<.001) and that d' for the frontal orientation (M = 1.773, SE = .144) was lower than d' for the profile (M = 2.246, SE = .106, p = .014), 30° (M = 2.339, SE = .094, p = .006), and 60° orientation (M = 2.237, SE = .060, p = .001), as predicted.

**Figure 4:**
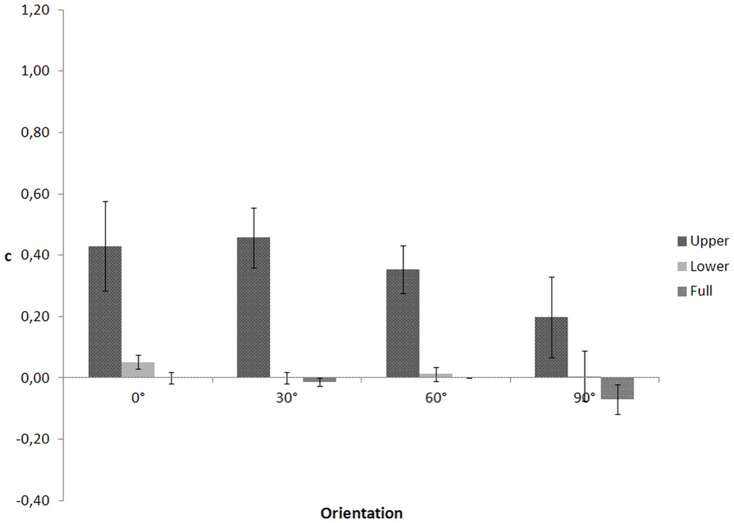
c as a function of visible body part and figure orientation for the walking action in Experiment 2.

In an ANOVA on c (Fig. 4), Mauchly's tests indicated that the assumptions of sphericity for body part (χ2(2)  = 6.941, p = .031), orientation (χ2(5)  = 12.352, p = .031), and the interaction between body part and orientation (χ2(20)  = 83.587, p<.001) were violated. Degrees of freedom were corrected using Huynh-Feldt estimate of sphericity for body part (ε = .752), and Greenhouse-Geisser estimates for orientation (ε = .597) and the interaction (ε = .349). (One note is in order here. Sphericity within this context means equality between any pair of variances of differences. For instance, we have three body parts conditions and three possible pairs of variances of differences: variance of the difference between upper and lower vs. variance of the difference between upper and full, variance of the difference between upper and lower vs variance of the difference between lower and full, variance of the difference between lower and full vs. variance of the difference between upper and full. There at least two possible corrections for violations of sphericity: the Greenhouse-Geisser estimate (e^∧^) and the Huynh-Feldt estimate (e∼). e^∧^ is too conservative causing incorrect acceptance of the null hypothesis that sphericity does hold, when it does not (Type II error). e∼ is too liberal causing incorrect rejection of the null hypothesis that sphericity does hold, when it does (Type I error). Girden (1992 [46]; also see Barcikowski & Robey, 1984 [47]) recommends that when e^∧^>0.75 then the df should be corrected using e∼. If e ^∧^<0.75, or nothing is known about sphericity at all, then the conservative e^∧^ should be used to adjust the df.)

The ANOVA yielded a significant main effect of body part, F(1.503, 19.544)  = 38.659, p<.001. The main effect of orientation, F(1.790, 23.275)  = 1.452, p = .254 and the interaction effect between body part and orientation, F(2.092, 27.192)  = .597, p = .565 were not significant. Post-hoc comparisons showed that c values for the upper part conditions (M = .360, SE = .049) were higher than for the lower part (M = .018, SE = .026, p<.001), and full body conditions (M = −.021, SE = .013, p<.001). However, this was not observed in Experiment 1 (and neither in subsequent experiments). The reason for this discrepancy is unclear at present.

## Experiment 3

In Experiments 1 and 2, we observed that the perception of motion direction of a walker on the basis of the articulatory, relative motions of the limbs is mainly driven by the motion of the lower limbs. This indirectly supports the hypothesis of a life detector mechanism particularly sensitive to the movements of the legs. However, for other, less common, types of human locomotion the movements of the arms might become more informative. In Experiment 3, participants were presented with a crawling action instead of a walking action, again either showing the full body or only the upper or lower body. We expected that in this case not only the lower body but also the upper body would be diagnostic for the direction of articulation. In addition, the depth orientation of the actor was manipulated as in Experiment 2, again under the assumption that, as the orientation was further away from the most informative sagittal view, performance would deteriorate.

### Method

#### Participants

14 students of psychology at the KU Leuven (12 women, 2 men, M_age_ = 18.95, SD_age_ = 1.18 years) participated in this experiment. They did not take part in the previous experiments, had normal or corrected-to-normal vision, and were naïve to the purpose of the study.

#### Stimuli

Participants were presented with the crawling action of the action database of Vanrie and Verfaillie [12]. On each trial, the action was again presented in one of four possible depth orientations: 0° (sagittal), 30°, 60°, or 90° (frontal). Either the full crawler or only the upper or lower body part was shown.

#### Procedure and design

The procedure was the same as in Experiment 2. Participants had to indicate the perceived direction of articulatory movement of upper, lower, or full crawlers shown in different depth orientations. Two blocks of trials, each containing 120 trials (5 repetitions of 24 unique trials resulting from the manipulation of 2 directions of motion, 3 types of crawlers, and 4 orientations) were administered to each participant.

### Results and discussion

**Figure 5:**
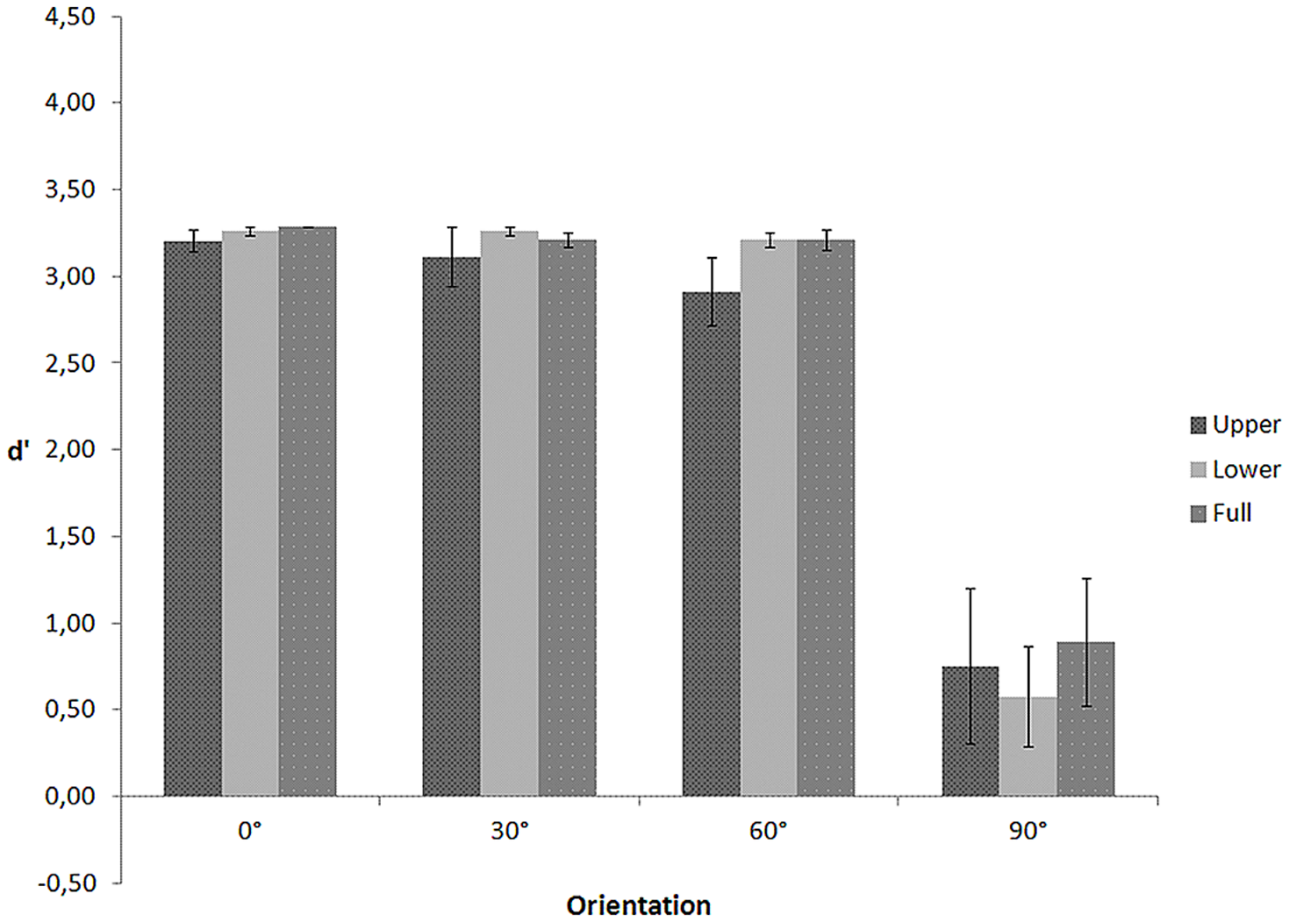
d' as a function of visible body part and figure orientation for the crawling action in Experiment 3.

A repeated measures ANOVA was performed on d' (see Fig. 5) with visible body part (upper, lower, or full) and depth orientation (0°, 30°, 60°, or 90°) as within-subjects variables. Because Mauchly's tests indicated that the assumptions of sphericity for body part (χ2(2)  = 8.842, p = .012), orientation (χ2(5)  = 42.807, p<.001), and the interaction between body part and orientation (χ2(20)  = 90.214, p<.001) were violated, degrees of freedom were corrected using Greenhouse-Geisser estimates of sphericity for body part (ε = .657), orientation (ε = .397), and the interaction (ε = .323). The ANOVA yielded a significant main effect of orientation, F(1.192, 15.501)  = 84.019, p<.001. Planned comparisons showed that d' for the frontal orientation (M = .742, SE = .257) was lower than for the profile (M = 3.253, SE = .021, p<.001), 30° (M = 3.197, SE = .058, p<.001), and 60° orientation (M = 3.111, SE = .068, p<.001). As predicted, the frontal view is less informative for determining the motion direction of the crawler. The main effect of body part, F(1.315, 17.090)  = .520, p = .529, and the interaction between body part and orientation, F(1.936, 25.170)  = .401, p = .667, were not significant. This suggests that, in contrast to walking, for crawling both the upper and the lower body part are diagnostic for determining the direction of articulatory motion.

**Figure 6:**
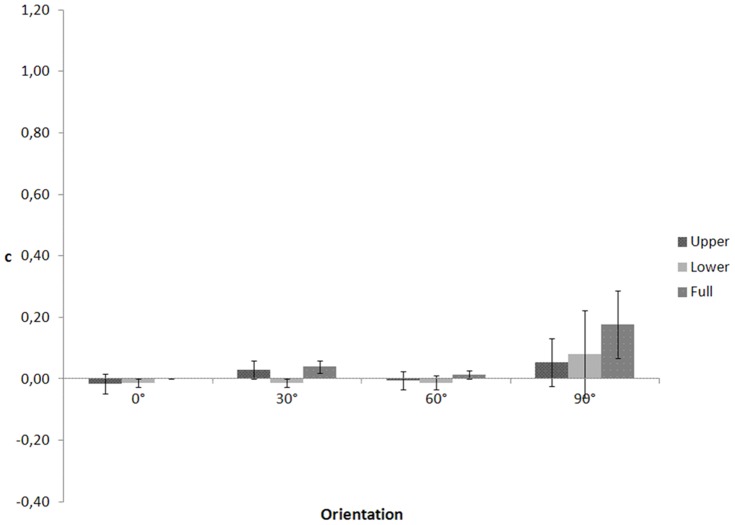
c as a function of visible body part and figure orientation for the crawling action in Experiment 3.

In an ANOVA on c, (Fig. 6) Mauchly's tests indicated that the assumptions of sphericity for orientation (χ2(5)  = 34.005, p<.001) and the interaction between body part and orientation (χ2(20)  = 98.790, p<.001) were violated. Therefore, degrees of freedom were corrected using Greenhouse-Geisser estimates of sphericity for orientation (ε = .406) and the interaction (ε = .377). The main effects of body part, F(2, 26)  = .762, p = .477, and orientation, F(1.218, 15.835)  = 1.964, p = .180, and the interaction between body part and orientation, F(2.262, 29.401)  = .254, p = .803, were not significant.

## Experiment 4

In Experiment 4, we presented participants with a (very uncommon) action for which the direction of articulatory motion probably is mainly perceived on the basis of the movements of the arms and the legs carry no useful information at all, namely hand walking. Participants again had to indicate the direction of articulatory motion and viewpoint and visible body part were manipulated as in the previous experiments. (Note that, because the actor is inverted, reference to upper and lower body parts becomes ambiguous now; we refer to the arms when we mention the upper body parts and to the legs when we mention the lower body parts.)

### Methods

#### Participants

13 female and 1 male new students of psychology at the KU Leuven (M_age_ = 18.59, SD_age_ = 0.41 years) and naïve with respect to the purpose of the study participated in this experiment.

#### Stimuli

A point-light human hand-walker was created using Autodesk 3ds Max 2012 [48] and a.csm file produced by Red Eye Studio [49]. The size of the stimulus was adapted to make it equivalent to the standard sizes of the stimuli from the Leuven Action Database used in the previous experiments.

#### Procedure and design

We used the same procedure and design as in Experiments 2 and 3.

### Results and discussion

**Figure 7:**
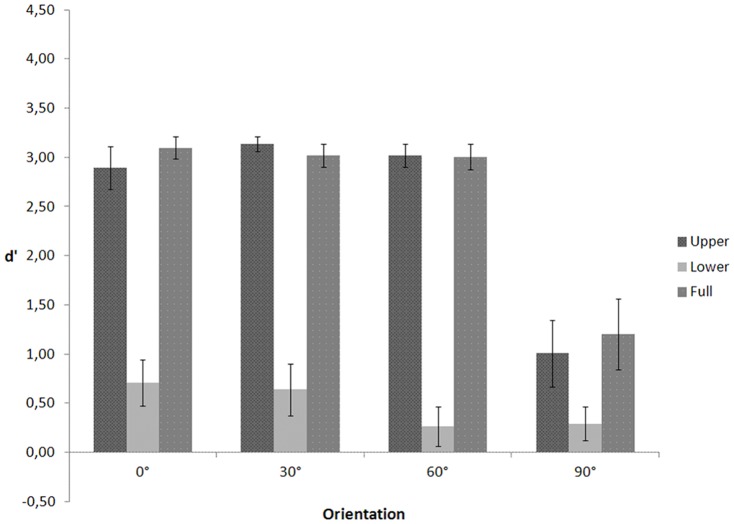
d' as a function of visible body part and figure orientation for the hand walking action in Experiment 4.

In a repeated measures ANOVA on d' (see Fig. 7) Mauchly's tests indicated that the assumptions of sphericity for body part (χ2(2)  = 16.402, p = <.001) and orientation (χ2(5)  = 19.592, p = .002) had been violated. Therefore, degrees of freedom were corrected using Greenhouse-Geisser estimates of sphericity for body part (ε = .573) and orientation (ε = .494). The ANOVA yielded a significant main effect of body part, F(1.146, 14.899)  = 164.069, p<.001, and orientation, F(1.481, 19.247)  = 19.377, p<.001, and a significant interaction effect between body part and orientation, F(6, 78)  = 8.762, p<.001. Planned comparisons showed that d' for the lower body part (M = .474, SE = .159) was lower than d' for the upper body part (M = 2.512, SE = .092) and the full body (M = 2.580, SE = .078, p<.001). As in the previous experiments, d' for the frontal orientation (M = .833, SE = .244) was lower than for the profile (M = 2.231, SE = .146, p = .001), 30° (M = 2.264, SE = .117, p<.001), and 60° orientation (M = 2.095, SE = .088, p<.001).

**Figure 8:**
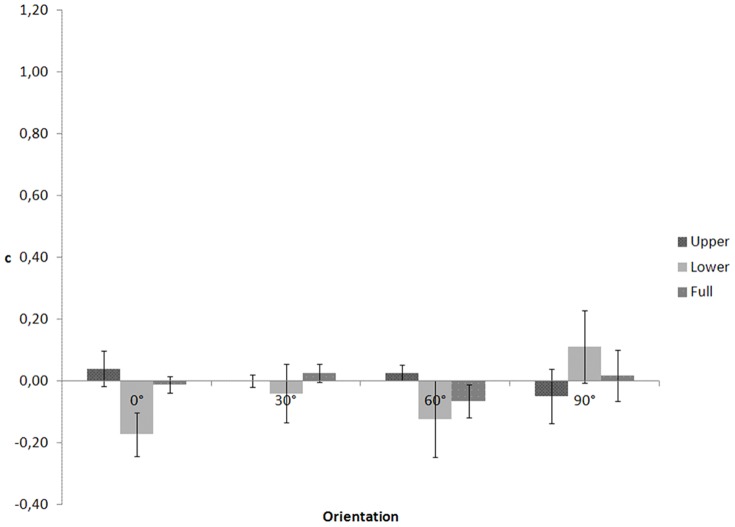
c as a function of visible body part and figure orientation for the hand walking action in Experiment 4.

**Figure 9:**
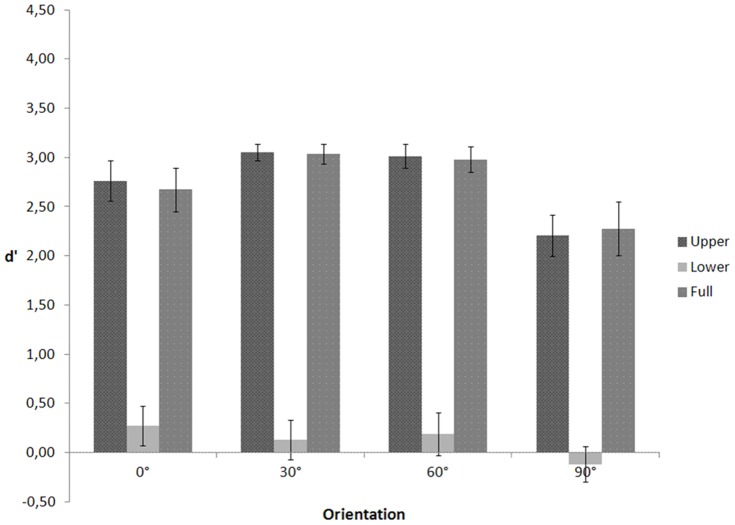
d' as a function of visible body part and figure orientation for the rowing action in Experiment 5.

In an ANOVA on c (Fig. 8), Mauchly's tests indicated that the assumptions of sphericity for body part (χ2(2)  = 12.865, p = .002) and the interaction between body part and orientation (χ2(20)  = 35.655, p = .021) had been violated. Therefore, degrees of freedom were corrected using Greenhouse-Geisser estimates of sphericity for body part (ε = .631) and Huynh-Feldt estimates of sphericity for the interaction (ε = .793) (Fig. 9). The main effects of body part, F(1.206, 15.684)  = .638, p = .808, and orientation, F(3, 39)  = .862, p = .187, and the interaction between body part and orientation, F(4.756, 61.825)  = 1.447, p = .471, were not significant.

## Experiment 5

The purpose of the final experiment was to study perception of motion direction in a rowing action, an action in which (in contrast to walking and crawling) the upper limbs probably are more diagnostic than the lower limbs. Participants again had to indicate the direction of articulatory motion and the figure's depth orientation and the visible body part was manipulated.

Rowing diverges from the actions presented in the previous four experiments in several respects. First, forward direction of motion is opposite to the facing direction of the mover. Second, like in hand walking, motion direction probably is best signalled by the movements of the upper limbs (and not by the lower limbs), but in contrast to hand walking the limbs produce a lot of (albeit non-diagnostic) motion energy. Third, and maybe most importantly, unlike walking, crawling, and hand walking (intransitive motion), direct contact of the limbs with the ground surface is not the source of translatory motion of the body as a whole. Rather, forward motion originates from moving (in this case rotating) an external device (in this case peddles) indirectly acting on the external environment (transitive motion). (Another typical example of a transitive action including rotatory motion of a tool is cycling.)

### Method

#### Participants

14 new students of psychology at the KU Leuven (11 women, 3 men, M_age_ = 18.93, SD_age_ = 1.01 years) participated.

#### Stimuli

We selected the rowing action from the point-light action database from Vanrie and Verfaillie [12]. The action displays a man rowing in a stationary standard boat (i.e., the translational motion component was removed). The action was again presented in one of four possible depth orientations: 0° (sagittal), 30°, 60°, or 90° (frontal) and either the full rower or only the upper or lower body part was shown.

#### Procedure

The procedure was the same as in Experiments 2, 3, and 4. Each participant was administered two blocks of 120 trials (each block containing 5 trials in each of 24 conditions (2 directions of motion ×3 types of rowers ×4 orientations)).

### Results and discussion

Sensitivity values (d') as a function of visible body part and depth orientation are shown in Fig. 9. In a repeated measures ANOVA, Mauchly's tests indicated that the assumptions of sphericity for body part (χ2(2)  = 7.514, p<.023) and the interaction between body part and orientation (χ2(20)  = 48.920, p<.001) had been violated. Therefore, degrees of freedom were corrected using Greenhouse-Geisser estimates of sphericity for body part (ε = .682) and the interaction (ε = .529). The main effect of body part, F(1.365, 17.743)  = 276.573, p<.001, and of orientation, F(3, 39)  = 10.082, p<.001, were statistically significant. The interaction between body part and orientation, F(3.174, 41.256)  = 1.172, p = .333, was not significant. Planned comparisons showed that d' for the lower body part (M = .119, SE = .136) was lower than d' for the upper body part (M = 2.759, SE = .114) and d' for the full body (M = 2.741, SE = .149, p<.001). When the rower was seen in a frontal orientation d' (M = 1.455, SE = .158) was lower than when seen in a profile orientation (M = 1.903, SE = .166, p = .008), 30° orientation (M = 2.073, SE = .099, p = .001), or 60° orientation (M = 2.061, SE = .110, p<.001). This confirms the prediction that, for a rowing action, the direction of articulatory motion is mainly driven by the motion of the upper limbs and that the frontal view is less diagnostic than the other views.

**Figure 10:**
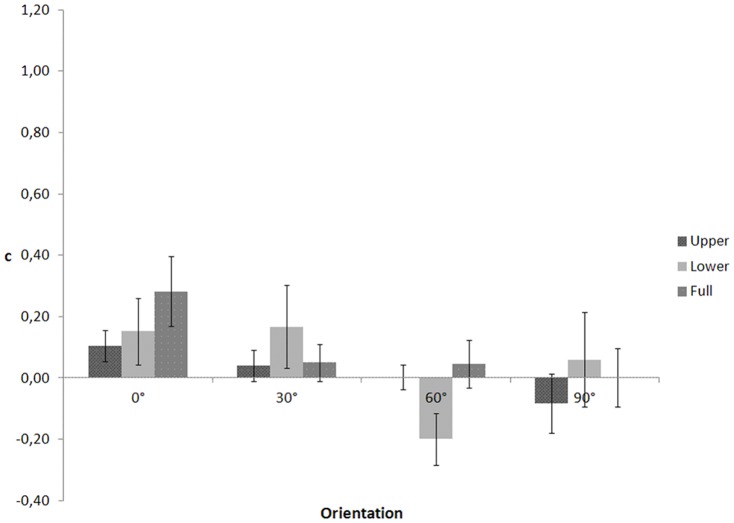
c as a function of visible body part and figure orientation for the rowing action in Experiment 5.

In an ANOVA on c, (Fig. 10) Mauchly's tests indicated that the assumptions of sphericity for the interaction between body part and orientation (χ2(20)  = 35.349, p = .022) had been violated. Therefore, degrees of freedom for the interaction were corrected (ε = .787). The ANOVA yielded a significant main effect of orientation, F(3, 39)  = 4.377, p = .015. Apparently, when confronted with the less informative frontal orientation, participants showed a tendency to interpret the low-informative stimulus as moving forward rather than backward. The main effect of body part, F(2, 26)  = .624, p = .544, and the interaction between body part and orientation, F(4.721, 61.368)  = 1.131, p = .353, were not significant.

## General Discussion

The main purpose of the present study was to examine the perception of motion direction of point-light actions on the basis of the articulatory relative motions of the limbs. Most importantly, this question was addressed not only for the action of walking (as done in previous studies), but also for the actions of crawling, hand walking, and rowing. In addition, we manipulated the diagnosticity of the information available for detection of the direction of articulatory motion by varying the visible body part (upper, lower, vs. full body) and the viewpoint from which the action was seen.

We observed that sensitivity for the direction of articulatory motion varies with the action: for walking the lower limbs are most diagnostic, for crawling both the upper and the lower limbs, for hand walking the upper limbs, and for rowing the upper limbs. The main conclusion is that, not surprisingly, the limbs that make contact to the ground surface and therefore enable locomotion are critical for the perception of direction of articulation.

In addition, performance in general was worst for the least informative frontal orientation [13]. This makes sense given that, for the actions used in the present study, the limbs almost entirely move in the midsagittal plane, i.e., in planes symmetrical to the facing direction of the moving figure [50], [51]. Information on motion direction in these planes obviously is weaker as the figure approaches the frontal/back view.

The next logical step in this research project is to try to relate sensitivity to quantitative stimulus characteristics for different actions in different depth orientations. We assume that for the task of classifying the horizontal direction of articulation of point-light figures, the asymmetry in the direction of motion of the dots is the critical information that produces performance. More specifically, we hypothesize that the relevant kinematic properties associated to this asymmetry are asymmetries between horizontal forward and backward motion in duration, velocity, and/or acceleration of individual or groups of dots. For instance, in the case of the action of walking, the upper body part in frontal orientation might be poorly informative because of the lack of asymmetry between forward and backward motion characteristics. A way to measure this (a)symmetry is calculating the absolute value of the difference between the duration, velocity, or acceleration when moving forwards and their correspondents when moving backwards and averaging these measures over dots. In Table 1 we present the Pearson correlation between sensitivity on the one hand and the asymmetry between forward and backward motion for duration, velocity, and acceleration for the upper and lower body in the different actions (averaged across orientations) on the other hand. As expected, for walkers performance is driven mostly by perception of the lower limbs, for crawlers by both upper and lower limbs, for handwalkers by upper limbs, and for rowers by upper limbs as well. We are currently investigating the relation between sensitivity and stimulus characteristics in more detail.

**Table 1:** Pearson correlation between sensitivity on the one hand and the asymmetry between forward and backward motion for duration, velocity, and acceleration for the upper and lower body in the different actions on the other hand.

	Walker		Crawler		Handwalker			Rower	
	Upper	Lower	Upper	Lower		Upper	Lower	Upper	Lower
duration	0.175	0.434	0.387	0.773***		0.702***	0.109	0.582***	−0.138
velocity	−0.156	0.472	−0.376	−0.719***		−0.600**	−0.304*	−0.301*	0.228
acceleration	0.199	0.553***	0.507***	0.730***		0.673***	−0.050	0.531***	−0.118

*p<.05.

**p<.01.

***p<.001.

Note that, despite the long stimulus duration relative to other studies, d' was still low for the upper body (for walking) or lower body (for hand walking). The long duration could have hidden differences between upper and lower limbs in the crawling condition, as d' was very high in all but the 90 degree orientation condition. Future studies should examine the effects of stimulus duration on the use of different body parts in biological motion perception, especially given our interest in the possibility of an innate visual filter that is tuned to quickly and automatically detect actions.

As mentioned in the Introduction, Troje and colleagues suggested that a specialized life detector is activated during the perception of animate locomotion. Obviously, the present data do not provide *direct* empirical evidence for the existence of a life detector mechanism. On the other hand, our findings are not in disagreement with such an account. Moreover, and this is speculative, if such a mechanism indeed underlies the behavior of the participants in the present experiments, the observations suggest an extension of the idea of a life detector as originally conceived of. Indeed, it is not the case that the lower limbs necessarily and exclusively carry the most useful information. Instead, for other actions (like crawling) both the upper and lower limbs are diagnostic for motion direction or (like hand walking) the lower limbs are not informative at all and information on the upper limbs drive the decision on the direction of motion. Apparently, and almost trivially, the most important factor is which limbs are directly responsible for direct contact with – and therefore locomotion – across the ground surface: the feet in the case of walking, the hands in the case of hand walking, and the hands and the feet in the case of crawling. Whether there is also something “special” about animate motions in the case of transitive actions (in which locomotion results from physical action on a tool) is an open question.
